# Grain Germination Changes the Profile of Phenolic Compounds and Benzoxazinoids in Wheat: A Study on Hard and Soft Cultivars

**DOI:** 10.3390/molecules28020721

**Published:** 2023-01-11

**Authors:** Julia Baranzelli, Sabrina Somacal, Camila Sant’Anna Monteiro, Renius de Oliveira Mello, Eliseu Rodrigues, Osmar Damian Prestes, Rosalía López-Ruiz, Antonia Garrido Frenich, Roberto Romero-González, Martha Zavariz de Miranda, Tatiana Emanuelli

**Affiliations:** 1 Department of Food Technology and Science, Center of Rural Sciences, Federal University of Santa Maria, Santa Maria 97105-900, Rio Grande do Sul, Brazil; 2 Department of Food Science, Federal University of Rio Grande do Sul, Porto Alegre 91501-970, Rio Grande do Sul, Brazil; 3 Department of Chemistry, Center of Natural and Exact Sciences, Federal University of Santa Maria, Santa Maria 97105-900, Rio Grande do Sul, Brazil; 4 Research Group ‘Analytical Chemistry of Contaminants’, Department of Chemistry and Physics, Research Center for Mediterranean Intensive Agrosystems and Agri-Food Biotechnology (CIAIMBITAL), University of Almeria, 04120 Almeria, Spain; 5 Grain Quality Laboratory, Brazilian Agricultural Research Corporation-Embrapa Trigo, Passo Fundo 99050-970, Rio Grande do Sul, Brazil

**Keywords:** cereal, pre-harvest sprouting, bioactive compounds

## Abstract

Pre-harvest sprouting is a frequent problem for wheat culture that can be simulated by laboratory-based germination. Despite reducing baking properties, wheat sprouting has been shown to increase the bioavailability of some nutrients. It was investigated whether wheat cultivars bearing distinct grain texture characteristics (BRS Guaraim, soft vs. BRS Marcante, hard texture) would have different behavior in terms of the changes in phytochemical compounds during germination. Using LC-Q-TOF-MS, higher contents of benzoxazinoids and flavonoids were found in the hard cultivar than in the soft one. Free phytochemicals, mainly benzoxazinoids, increased during germination in both cultivars. Before germination, soft and hard cultivars had a similar profile of matrix-bound phytochemicals, but during germination, these compounds have been shown to decrease only in the hard-texture cultivar, due to decreased levels of phenolic acids (*trans*-ferulic acid) and flavonoids (apigenin) that were bound to the cell wall through ester-type bonds. These findings confirm the hypothesis that hard and soft wheat cultivars have distinct behavior during germination concerning the changes in phytochemical compounds, namely the matrix-bound compounds. In addition, germination has been shown to remarkably increase the content of benzoxazinoids and the antioxidant capacity, which could bring a health-beneficial appeal for pre-harvested sprouted grains.

## 1. Introduction

Wheat (*Triticum aestivum* L.) is among the most-produced cereals in the world. Wheat production is affected by climate changes [1]. Under conditions of high humidity and temperature, cereal crops suffer a process of natural germination before harvest. This process, which is known as pre-harvest sprouting, reduces grain quality and generates economic losses worldwide [1]. Sprouted wheat is usually discarded due to the loss of its baking properties [2,3,4] or it can be destined for uses other than baking [5,6], such as for feed or industrial uses (production of starch, vital gluten, furfural, ethanol, etc.).

In contrast, grain germination has been also used as a natural method of biological processing to improve the nutritional, functional and sensory properties of grains along with increasing micronutrient content [7]. Grain germination can increase the content of health-beneficial phytochemicals, such as phenolic compounds [8,9] and benzoxazinoids [10]. Phenolic compounds have antioxidant, antidiabetic and antitumor properties, and have been shown to prevent cardiovascular diseases [11]. Benzoxazinoids exhibit antimicrobial properties, act as central nervous system stimulators, immunoregulators, appetite inhibitors and body weight reducers [10].

Phenolic compounds are found in the form of glycosides linked to different sugar fractions, or in other forms linked to organic acids, amines, lipids, carbohydrates and other phenols, mainly phenolic acids [12]. Cereal phenolic acids occur in free (~25%) or bound (~75%) forms [13], being linked through ester or ether bonds to cell wall polysaccharides [14].

Benzoxazinoids are a class of natural products that are widely distributed in cereals, being concentrated in the cover layer of grains (pericarp) [15]. They can be divided into three groups, namely hydroxamic acids, lactams and benzoxazolinones [16], and have been shown to be increased during the germination of some cereal grains [17], but their behavior during wheat germination remains unknown.

The phytochemical compounds found in the free state are easily extracted with conventional organic solvents [18], whereas those linked to sugars and proteins, or cell wall structures, require very hard conditions for their extraction, such as acid or alkaline hydrolysis [19,20]. Grain germination is a physiological process that can trigger both the hydrolysis of matrix-bound phytochemical compounds and the formation of new compounds [3,21,22].

Therefore, wheat that is naturally germinated before harvest could have a higher content of phytochemical compounds, which would improve its functional quality and, thus, add value to this product. In fact, germination can increase the bioavailability of nutrients, such as vitamins, bioelements and other biologically active substances, due to the partial hydrolysis of starch, proteins, hemicelluloses and celluloses. In this process, hydrolytic enzymes are activated (endohydrolase such as α and β-amylases, proteases, diphenoloxidase and catalase) that break down starch, fibers and proteins, and lead to an increase in the number of digestible compounds along with an improvement in functional properties [23]. Laboratory-based germination that simulates field humidity and temperature conditions can be used as a model to study the influence of pre-harvest sprouting on wheat grain properties [2,5,6].

Another important property beneficial to health is the antioxidant capacity of cereals. Antioxidant compounds protect cells against oxidative stress caused by reactive species [23]. The main compounds responsible for such effects in cereals are vitamins, sterols, and phenolic compounds. They all contribute to some extent to the antioxidant properties and are affected in different ways by germination. Phenolic acids have potential antioxidant properties due to the presence of an aromatic phenolic ring. Its antioxidant properties are explained by the donation of electrons and the transfer of the hydrogen atom to free radicals. They act as free radical scavengers, reducing agents and inhibitors of singlet oxygen formation. Benzoxazinoids also exhibit antioxidant properties that are related to the presence of a hydroxyl group attached to the heterocyclic nitrogen atom, mainly in hydroxamic acids [24].

Wheat cultivars exhibit physiological differences and can be classified according to the grain hardness (method 55-31.01, AACCI, [25]). Wheat grains cultivated in Brazil are similar to the hard red spring wheat from the United States, but wheat production occurs only in the winter. BRS Marcante is a Brazilian wheat cultivar that has hard texture and exhibits high values of grain hardness index (GHI > 67), gluten strength (W > 275 × 10^−4^ J) [26], dough development time (DDT > 21.5 min) and stability (>30.2 min) [2], being indicated mainly to produce different kinds of bread and pasta. In contrast, BRS Guaraim has much lower values for these markers, exhibiting a soft grain texture (GHI < 46), W (<206 × 10^−4^ J) and DDT (<4.1 min) [27] and is more suitable for cake and cookie production.

The hypothesis of this study is that wheat cultivars bearing distinct wheat grain texture characteristics would have different behavior in terms of the changes in phytochemical compounds during germination. In this sense, the objective of this study was to investigate the effect of germination on the profile of phytochemical compounds and antioxidant capacity of Brazilian wheat cultivars that have soft or hard grain texture.

## 2. Results and Discussion

### 2.1. Grain Characteristics, Alveography and Germination Follow-Up in Wheat Cultivars

The wheat cultivars selected for the study have different technological characteristics. BRS Marcante cultivar presents higher values for grain texture (GHI), GFN, hectoliter weight and thousand kernel weight compared to BRS Guaraim (111%, 62%, 8% and 12% higher, respectively; Supplementary materials, Table S1). Additionally, the alveograph data were also higher for BRS Marcante cultivar, as observed for gluten strength, tenacity and tenacity/extensibility ratio parameters (164%, 101% and 49% higher, respectively; Supplementary materials, Table S1), in relation to BRS Guaraim cultivar.

Grain hardness is the single most important trait in determining technological properties and end-use quality of wheat products. Hard-textured wheats have more glutenins, whereas soft-textured wheats have more gliadins [28]. The greater content of glutenins leads to increased gluten strength, elasticity and extensibility of the dough.

The germination process conditions used in this study resulted in small sprouts at 24 h (Supplementary materials, Table S2). From 48 h onwards, the grains began to emit small rootlets that became larger at 72 h, but the emergence of the seedling did not occur (Supplementary materials, Table S2). Germination is characterized by the activation of enzymes, including α-amylase, which is responsible for the degradation of starch into sugars that provide energy for the embryo’s growth [29]. GFN was assessed to characterize changes in amylase activity during wheat germination (Table 1). Before germination, GFN was higher for BRS Marcante than BRS Guaraim (*p* < 0.05; Table 1), indicating lower amylase activity for BRS Marcante.

As expected, the germination process decreased GFN, indicating increased amylolytic activity, which reduces starch content and gelatinization capacity [30]. The increase in amylolytic activity was faster for BRS Marcante than BRS Guaraim, as indicated by the lower GFN values at 24 and 48 h (*p* < 0.05; Table 1). The increase in grain moisture during germination causes a gradual decrease in the falling number and in the grain hardness index [31]. This causes the texture of the durum wheat grain to become softer after germination, possibly due to a decrease in the starch crystallinity [32]. This alteration facilitates the enzymatic attack and decrease of GFN with the advance of germination.

### 2.2. Tentative Identification of Phytochemical Compounds

Thirty-two phytochemical compounds, comprising amino acids, phenolic acids, flavonoids and benzoxazinoids, were identified in BRS Marcante and BRS Guaraim wheat cultivars based on the data provided by LC-Orbitrap-MS and LC-Q-TOF-MS analysis (Supplementary materials, Figure S1 and Table S3). Eighteen compounds were in the free state, whereas fourteen compounds were extracted only after alkaline hydrolysis (eight compounds), indicating that they were linked to the cell wall or simple sugars through ester-type bonds, or after acid hydrolysis (six compounds), indicating that they were linked to polysaccharides through ether-type glycosidic bonds [14]. Previous studies have already reported the presence of phenolic compounds and flavonoids [33,34,35,36], amino acids [37] and benzoxazinoids [36,38,39] in wheat.

The greatest diversity of compounds was identified in the free phytochemical fraction. Amino acids were identified in LC-Q-TOF-MS analysis in the positive ionization mode. Phenylalanine (RT 6.3 min) showed a protonated MS spectrum [M + H]^+^ at *m/z* 166 and MS^2^ fragments at *m/z* 120 e 103. The main fragmentation pathway of phenylalanine starts from the loss of H_2_O + CO to form a fragment ion at *m/z* 120.0851, and a fragment ion at *m/z* 103.0590 was formed by the additional loss of NH_3_. Tryptophan showed a protonated MS spectrum [M + H]^+^ at *m/z* 205 and MS^2^ fragments at *m/z* 118, 143, 146, 144 and 115. The fragmentation product *m/z* 146.0636 was obtained after the loss of NH_3_ and CH_2_CO. This fragment further dissociated to form the fragmented ion at *m/z* 118.0717 after the loss of CO. The fragment at *m/z* 144.0827 was formed after the loss of NH_3_ and CO_2_, and the dissociation of ^•^H forms the fragment *m/z* 143.0776. The fragmented ion at *m/z* 115.0588 was formed after the sequential losses of NH_3_, H_2_O, CO and HCN. In addition, amino acids showed MS spectrum and MS^2^ fragmentation patterns similar to previously reported data [37].

Among benzoxazinoids, two compounds that belong to the hydroxamic acids subclass were identified, namely DIBOA-hex-hex and DIMBOA-hex-hex. At 17.2 min, the compound was tentatively identified as DIBOA-hex-hex (MW = 505). In the negative ionization mode, the MS spectrum showed the deprotonated molecule [M−H]^−^ at *m/z* 504 and MS^2^ fragment at *m/z* 162 [M−H−342]^−^, corresponding to the loss of two hexose molecules (C_12_H_22_O_11_), and at *m/z* 134 [M −H−370]^−^, corresponding to the sequential loss of CO. At 24.3 min, the compound was tentatively identified as DIMBOA-hex-hex (MW = 535). In the negative ionization mode, the MS spectrum showed the deprotonated molecule [M−H]^−^ at *m/z* 534 and MS^2^ fragment at *m/z* 192 [M−H−342]^−^, corresponding to the loss of two hexose molecules, at *m/z* 164 [M−H−370]^−^, corresponding to the sequential loss of CO, and at *m/z* 149 [M−H−385]^−^, corresponding to the sequential loss of ^•^CH_3_. In addition, benzoxazinoids exhibited MS spectrum and MS^2^ fragmentation patterns similar to previously reported data [36,39].

Phenolic acids and some flavonoids from the free fraction were identified by comparison with standards and confirmed using LC-Orbitrap-MS. Four apigenin derivatives were identified using LC-Q-TOF-MS in the negative ionization mode. Compounds eluted at 38.5 and 42.8 min were identified as apigenin-hex-pent I e II (MW = 564). The MS spectrum showed the deprotonated molecule [M−H]^−^ at *m/z* 563 and fragment in MS^2^ at *m/z* 443 [M−H−120]^−^, corresponding to the loss of C_4_H_8_O_4_, at *m/z* 353 [M−H−210]^−^, corresponding to the loss of C_7_H_14_O_7_, and at *m/z* 383 [M−H−90]^−^, corresponding to the loss of C_8_H_14_O_6_. Moreover, the MS spectrum and MS^2^ fragmentation patterns were similar to previously reported data [40]. The compounds eluted at 52.4 and 53.1 min were identified as apigenin-hex-hex-hex I e II (MW = 770). In the negative ionization mode, the MS spectrum showed the deprotonated molecule [M−H]^−^ at *m/z* 769 and fragment in MS^2^ at *m/z* 425 [M−H−344]^−^, corresponding to the loss of a molecule of C_6_H_10_O_7_ (194) and a molecule of C_5_H_10_O_5_ (150), and the dissociation of ^•^H forms the fragment *m/z* 426.0868. At *m/z* 545 [M−H−224]^−^, corresponding to the loss of C_9_H_12_O_3_ (168) and two molecules of CO (56), the dissociation of ^•^H forms the fragment *m/z* 546.1238. Moreover, MS spectrum and MS^2^ fragmentation patterns were similar to previously reported data for apigenin-hex-hex-hex [41,42].

The fraction of phytochemicals extracted through alkaline and acid hydrolysis, which comprises matrix-bound compounds, showed less diversity compared to the free fraction, being composed mainly of phenolic acids and a flavonoid. All compounds were identified by comparison to an authentic standard using LC-Orbitrap-MS analysis. The presence of p-coumaric acid, *trans* and *cis*-ferulic acid were also confirmed using LC-Q-TOF-MS analysis.

The compound eluted at 27.8 min was tentatively identified as *p*-coumaric acid (MW = 164). In the negative ionization mode, the MS spectrum showed the deprotonated molecule [M−H]^−^ at *m/z* 163 and fragment in MS^2^ at *m/z* 119 [M−H−44]^−^, corresponding to the loss of CO_2_. Moreover, the MS spectrum and MS^2^ fragmentation patterns were similar to previously reported data for *p*-coumaric acid [36].

The compounds eluted at 35.7 and 36.7 min were tentatively identified as *trans* and *cis*-ferulic acid (MW = 194). In the negative ionization mode, the MS spectrum showed the deprotonated molecule [M−H]^−^ at *m/z* 193. Ferulic acid produces a typical negative fragment in MS^2^ at *m/z* 133 [M−H−60]^−^ from demethylation and decarboxylation, corresponding to the loss of C_2_H_4_O_2_, and the dissociation of ^•^H forms the fragment *m/z* 134.0368 [36].

Some peaks could not be identified, but one of them, which was the most abundant in the extract obtained after acid hydrolysis (Figure S1c), had MS characteristics that are not compatible with phenolic or benzoxazinoid compounds.

### 2.3. Changes in the Phytochemical Profile of Wheat during Germination

The phytochemical profiles of BRS Guaraim and BRS Marcante across 72 h of germination are shown in Table 2 and Table 3. Before germination, most phytochemicals of BRS Guaraim and BRS Marcante cultivars were bound to the grain matrix (64% released by alkaline hydrolysis and 8% released by acid hydrolysis), being composed mainly of phenolic acids, followed by flavonoids (Table 3). *Trans*-Ferulic acid was the major single compound found in the wheat grains (46–48% of the total phytochemicals) before germination followed by *p*-coumaric acid (9–12% of total phytochemicals), as previously reported [22,43,44]. The fraction of free phytochemicals amounted to 28% of total phytochemicals from wheat, and was mainly composed of flavonoids, followed by benzoxazinoids and a minor content of phenolic acids (Table 2). Flavonoids mainly occur in C-glycoside forms [42], and glycosylated apigenins were the main flavonoid structures found (Table 2 and Table 3). Benzoxazinoids amounted to almost 10% of free phytochemicals and DIBOA-hex-hex was the predominant form of benzoxazinoids in the wheat (Table 2), as previously reported [45,46].

Free phenolic compounds are generally synthesized in the endoplasmic reticulum and stored in the vacuole of plant cells [19]. Looking at the sum of free phenolic acids, BRS Marcante had the highest levels (*p* < 0.05), but no changes were detected during germination regardless of the cultivar (Table 2). Sinapic acid was the major free phenolic acid being found at higher levels in BRS Marcante than BRS Guaraim (82% higher, *p* < 0.05), and its content was not altered during germination (Table 2). Before germination, vanillic acid was found only in BRS Guaraim, but decreased with the advance of germination, while in BRS Marcante, it showed a trend of increasing (Table 2).

DIBOA-hex-hex was the major benzoxazinoid in both cultivars and was 1.8-fold higher in BRS Marcante than in BRS Guaraim, whereas DIMBOA-hex-hex did not differ between cultivars (Table 2). Previous studies have already reported DIBOA dihexoside as the main benzoxazinoid found in wheat [41,45,47], but benzoxazinoid behavior during wheat germination was still unknown. Benzoxazinoids increased with the advance of germination (Table 2), reaching the highest levels at 72 h (480% higher value than at 0 h; Table 2). This finding is in line with previous reports showing the highest levels of benzoxazinoids in the sprouts of other cereals such as rye [17]. Benzoxazinoids are concentrated in the bran and germ [10], fractions which are mainly involved in germination.

Flavonoids were the major class of compounds in the free phytochemical fraction (~73% of free phytochemicals). Apigenins hex-pent II, hex-hex-hex I and hex-hex-hex II were higher in BRS Marcante than in BRS Guaraim, and this made the sum of flavonoids also higher in BRS Marcante (23% higher). Although most free flavonoids did not change during germination, apigenin (aglycone form) showed a U-shaped behavior that was not affected by cultivar, being decreased up to 48 h, followed by an increase at 72 h of germination (Table 2). BRS Guaraim showed a similar U-shaped behavior for apigenin hex-pent I that was not found in BRS Marcante (*p* < 0.05; Table 2). The cause of these changes may be related to the binding of apigenin aglycone with other structures, even forming glycosylated apigenins, or the release of cell wall bound apigenins or sugars and proteins [48]. De novo synthesis may also contribute to some of these changes [47]. The sum of free phytochemical compounds, which was 1.3-fold higher in BRS Marcante than BRS Guaraim, was remarkably increased during germination (48 h onwards, *p* < 0.05; Table 2).

Concerning the bound phytochemical compounds that are released by alkaline hydrolysis, there was no difference between cultivars in the sum of bound phenolic acids, but the content of sinapic acid released by alkaline hydrolysis was higher in BRS Marcante than BRS Guaraim (16% higher content, Table 3). Independent of the cultivar, there was a bell-shaped behavior for sinapic acid content during germination, with peak values at 48h of germination. Bound 4-hydroxybenzoic acid released by alkaline hydrolysis was linearly increased with the advance of germination in BRS Guaraim, while BRS Marcante showed a bell-shaped behavior with peak values at 48 h of germination (*p* < 0.05; Table 3). Bound vanillic acid released by alkaline hydrolysis, which was not detected in any wheat cultivar before germination, was found only in germinated grains of BRS Guaraim and exhibited a bell-shaped behavior with peak values at 48 h (Table 3). In the free fraction of this cultivar, it appeared only before germination. Free vanillic acid of BRS Guaraim may have been linked to complex cell wall structures through ester-type bonds during the beginning of germination, explaining the disappearance of the free form and its appearance after 48 h in the fraction released after alkaline hydrolysis [14]. Bound *p*-coumaric acid that was released by alkaline hydrolysis did not differ between cultivars or germination times (Table 3). *trans*-Ferulic acid was the major component (70%) among the bound phytochemicals released after alkaline hydrolysis (Table 3). *trans*-Ferulic acid and the sum of phenolic acids released by alkaline hydrolysis had a U-shaped behavior throughout germination in BRS Marcante, but not in BRS Guaraim (Table 3). The lowest content of *trans*-ferulic acid and of the sum of phenolic acids was found for BRS Marcante at 48 h, resulting in 1.8-fold lower content than BRS Guaraim.

Apigenin was the only flavonoid quantified in the bound fraction released after alkaline hydrolysis. In BRS Marcante, apigenin underwent a significant decrease at 72 h of germination (Table 3), which was not observed in BRS Guaraim.

Although there was no difference in the sum of bound phytochemicals released by alkaline hydrolysis between cultivars, the level of this group of compounds decreased in BRS Marcante from 48 h onwards, resulting in lower levels than BRS Guaraim (41% lower levels, Table 3). One explanation for this behavior is the release of compounds bound to the cell wall during germination, which increases the content of free compounds [48]. Accordingly, the sum of free phenolic compounds increased during germination.

In the fraction of bound compounds that were released by acid hydrolysis, only *p*-coumaric acid was identified (Table 3). There was a significant cultivar vs. germination time interaction in the levels of *p*-coumaric acid released by acid hydrolysis, resulting in higher levels for BRS Marcante than BRS Guaraim at 24 h of germination (25% higher levels, *p* < 0.05). However, no marked changes were observed in the content of this phenolic acid throughout germination. Kim, Kwak and Kim [44] found gallic, 4-hydroxybenzoic, vanillic, caffeic, syringic, ferulic and *p*-coumaric acids in the free and bound fractions of germinated and not-germinated wheat. Initially, they observed low levels followed by an increase with the advance of germination, and the highest levels were found at 72 h and 96 h of germination. Ferulic and vanillic acid had the highest increase with values up to 1.5-fold higher in germinated than in not-germinated grains. Most phenolic acids in wheat occur in the bound form and have been shown to increase with advancing germination, i.e., *p*-coumaric, ferulic and sinapic acids, likely through the decomposition of lignin and other chemical reactions [43,44].

### 2.4. Multivariate Analysis of Phytochemical Changes in during Wheat Germination

MANOVA using the likelihood ratio test (Wilks), Pillai, Hotelling–Lawley and Roy tests revealed that, when all dependent variables (phytochemical compounds) were combined in the analysis, there was a significant interaction effect of wheat cultivar vs. germination time (Supplementary materials, Table S4). This finding confirms the hypothesis that the soft and hard wheat cultivars evaluated showed distinct changes in free and bound phytochemical compounds during germination, and qualifies the data for the Principal Component Analysis (PCA).

**Figure 1:**
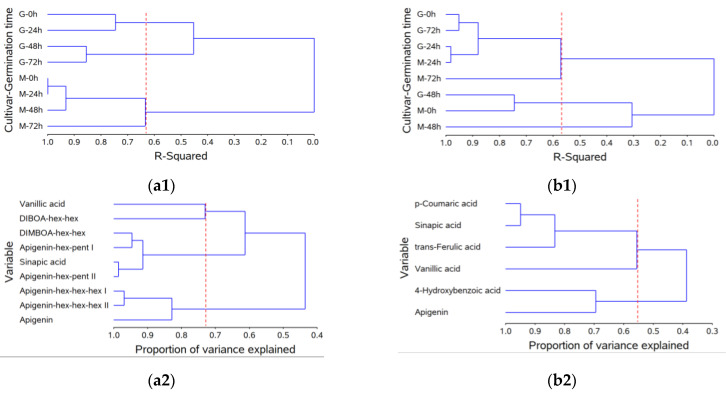
Dendrogram of wheat cultivars (G = Guaraim, M = Marcante) under different germination times (0, 24, 48 and 72 h; ordinate axis) in relation to the coefficient of determination (r2, abscissa axis) using Euclidean distance as a measure of dissimilarity and Ward’s agglomerative hierarchical algorithm as a clustering method for the free phytochemical fraction (**a1**) and for the bound phytochemical fraction that was released after alkaline hydrolysis (**b1**); and dendrogram of phytochemical content (mg/100 g, ordinate axis) in relation to the coefficient of determination (r2, abscissa axis) using the correlation matrix as a measure of similarity and the principal component as a clustering method for the free phytochemical fraction (**a2**) and for the bound phytochemical fraction that was released after alkaline hydrolysis (**b2**). **a1** = 63.3% and **a2** = 73.0%; **b1** = 57.1% and **b2** = 54.2%.

Cluster analysis further confirmed the distinct behavior of BRS Guaraim and BRS Marcante concerning the changes in phytochemicals during germination. Cluster analysis revealed that wheat cultivars were grouped into three groups according to the free phytochemical variables during germination (Figure 1(a1)), explaining 63.3% of the data variation. BRS Marcante was grouped into a single group regardless of the germination time (M-0 h, -24 h, -48 h and -72 h), whereas BRS Guaraim was divided into two groups: one for 0 and 24 h (G-0 h and -24 h) and the other for 48 h and 72 h (G-48 h and -72 h). Cluster analysis of the free phytochemical variables (Figure 1(a2)) also revealed three groups, which were able to explain 73% of the data variation. One of these groups included vanillic acid and DIBOA-hex-hex, another group was composed of DIMBOA-hex-hex, apigenin-hex-pent I and II and sinapic acid, and the last one was composed of apigenins hex-hex-hex I, II and aglycone.

The cluster analysis of wheat cultivars during germination according to the bound phytochemicals that were released after alkaline hydrolysis divided samples into three groups (57.1%) (Figure 1(b1)). The first group included BRS Guaraim 0, 24 and 72 h (G-0 h, -24 h and -72 h) and BRS Marcante 24 and 72 h (M-24 h and -72 h), the second group included BRS Guaraim 48 h and BRS Marcante 0 h (G -48 h and M-0 h) and the third group was represented only by BRS Marcante 48 h (M-48 h). The dependent variables (bound phytochemical compounds) of this analysis, in turn, were only divided into two groups (54.2%), one comprising *p*-coumaric, sinapic, *trans*-ferulic and vanillic acid, and the other comprising 4-hydroxybenzoic acid and apigenin (Figure 1(b2)).

**Figure 2:**
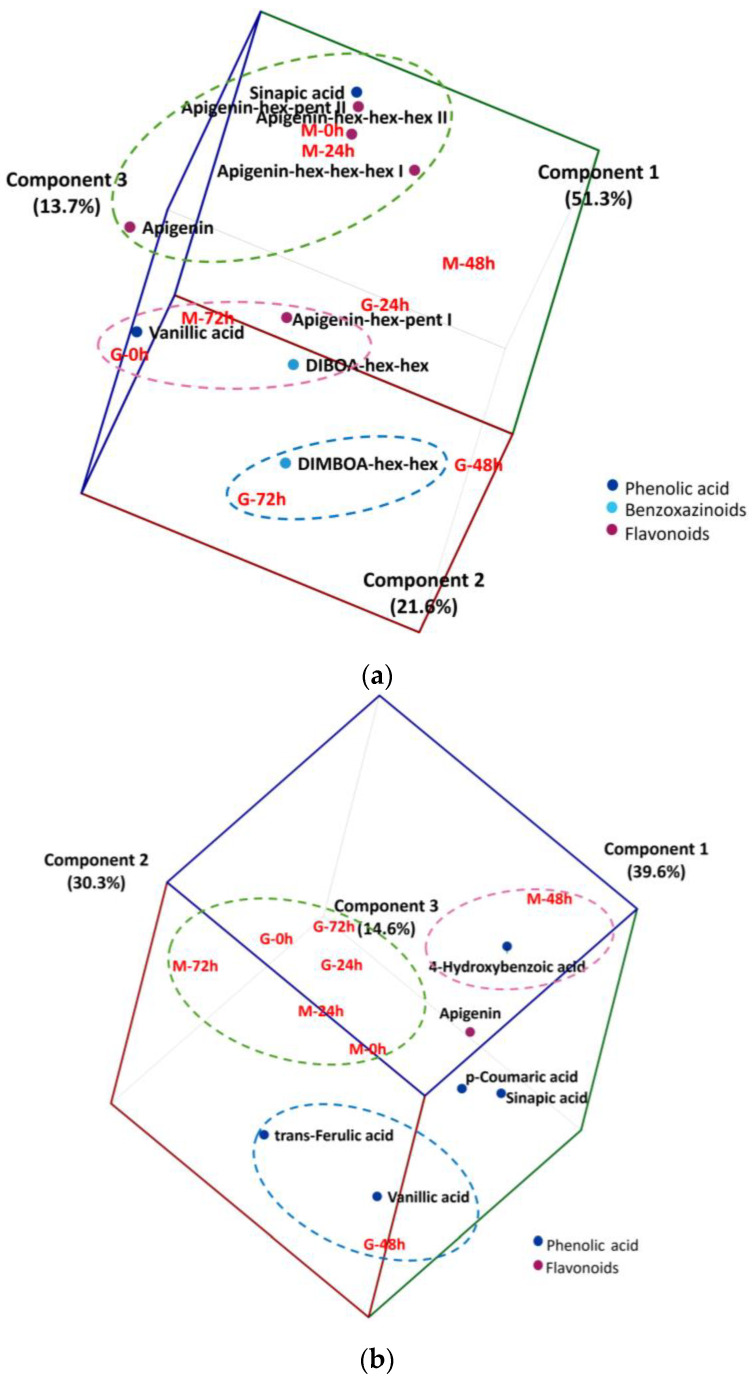
Three-dimensional biplot of wheat cultivars (G = Guaraim, M = Marcante) at different germination times (0, 24, 48 and 72 h) (scores) versus phytochemicals (loadings) in relation to the main components of principal component analysis for the free phytochemical fraction (**a**) and for the bound phytochemical fraction that was released after alkaline hydrolysis (**b**); a = 86.6% and b = 84.5%. Colored dashed ellipses indicate the proximity among samples and variables.

PCA was used as an exploratory analysis to verify if changes in the phytochemical compounds of free or bound fractions would allow the discrimination of wheat cultivars during germination (Figure 2). A biplot (cultivar and germination time vs. content of free phytochemicals, Figure 2a) confirmed the findings of cluster analysis and increased the proportion of explained variance to 86.6% using the first three principal components. Another biplot (cultivar and germination time vs. content of bound phytochemicals released after alkaline hydrolysis, Figure 2b) confirmed the findings of cluster analysis and increased the proportion of explained variance to 84.5% using the first three principal components.

BRS Marcante before germination and up to 24 h of germination was associated with high levels of some free phytochemicals, namely sinapic acid, apigenin and apigenin hex-pent II, and apigenin hex-hex-hex I and II (Figure 2a, green circle). With the progression of germination, BRS Marcante changed the profile of free phytochemical compounds, becoming associated with high levels of vanillic acid, apigenin hex-pent I and DIBOA hex-hex after 72 h of germination (Figure 2a, pink circle). On the other hand, BRS Guaraim was already associated with high levels of vanillic acid, apigenin hex-pent I and DIBOA hex-hex before germination (Figure 2a, pink circle), which were changed to a free phenolic profile associated with DIMBOA hex-hex after 72 h of germination (Figure 2a, blue circle).

Bound phenolic compounds are located mainly in the cell wall and are formed through the conjugation of free phenolic compounds with macromolecules such as cellulose and proteins [22]. Concerning the profile of bound phytochemicals that were released after alkaline hydrolysis, there was an association between BRS Guaraim and BRS Marcante samples at the start (0 and 24 h) and in the end of germination (72 h) (Figure 2b, green circle). However, at 48 h of germination there was a distinct profile of bound phytochemicals between cultivars. While BRS Marcante was associated with high levels of 4-hydroxybenzoic acid (pink circle), BRS Guaraim was associated with high levels of vanillic acid and *trans*-ferulic acid (blue circle; Figure 2b).

### 2.5. Antioxidant Capacity of Wheat Cultivars during Germination

**Figure 3:**
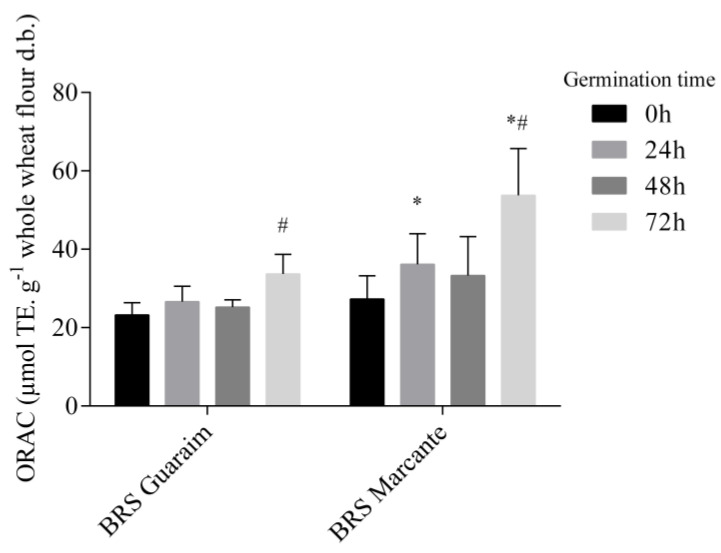
Changes in the oxygen radical absorbance capacity (ORAC) of the free phytochemical fraction of germinated wheat. # Significantly different (*p* < 0.05) from the same cultivar at 0 h. * Significantly different (*p* < 0.05) from BRS Guaraim at the same germination time. n = 6.

During germination, in addition to the hydrolysis of macronutrients such as protein [49] and starch (Table 1), phytochemical compounds may be synthesized, released or conjugated, which may result in changes in the antioxidant capacity (Figure 3). The antioxidant capacity is associated with the ability to scavenge free radicals, break radical chain reactions and chelate metals [50]. The oxygen radical absorbance assay was used to evaluate the antioxidant capacity of wheat grains. The antioxidant capacity was assessed only in the extract containing the free phytochemicals, which is expected to be solubilized during digestion and available for intestinal absorption. Although bound phytochemicals, namely bound phenolic compounds, have already been demonstrated to exert antioxidant effects in vivo [51,52], such effects depend on their interaction and biotransformation by gut microbiota, which was not investigated in the present study.

Although BRS Marcante had a higher content of free phytochemical compounds than BRS Guaraim (Table 2), the peroxyl radical scavenging capacity of both cultivars was similar before germination (Figure 3). The compounds that most contribute to this antioxidant activity are phenolic acids and flavonoids [53]. In both cultivars, there was an increase in the antioxidant capacity after 72 h of germination (*p* < 0.05), and this increase was higher for BRS Marcante, which had a higher antioxidant capacity than BRS Guaraim at 24 h (58% higher) and 72 h (41% higher; *p* < 0.05).

Many studies demonstrate increased antioxidant capacity in sprouted wheat [54,55,56,57,58] and generally attribute this to the release of bound phytochemicals. In contrast to the free phytochemical compounds that are soluble in conventional organic solvent, most phytochemical compounds of wheat are bound to cell wall components or sugars. These matrix-bound phytochemical compounds (BPC) are insoluble and, therefore, will not contribute to the antioxidant capacity assessed in the extracts of wheat grain. However, during food digestion, BPC will reach the colon, where they can be fermented by gut microbiota and exert nutraceutical properties [59]. In addition, BPC could be a substrate for metabolic transformation during grain germination. The diverse and complex structure of BPC makes it difficult to analyze these compounds. For this reason, alkaline and acid hydrolysis were used to release the small units of phytochemical compounds and obtain some insight into the type of chemical bonds responsible for their linkage to the food matrix. The total concentration of phytochemicals bound to the cell wall (ester-type bonds, alkaline hydrolysis) was 2.1- and 1.5-fold higher for BRS Guaraim and BRS Marcante, respectively, than the total concentration of free phytochemicals. Phytochemicals linked to simple sugars (ether-type glycosidic bonds, acid hydrolysis) were 3.6- and 4.5-fold lower, respectively. Of the total phytochemical compounds found in wheat, approximately 70% and 64% correspond to the total bound fraction, respectively, for BRS Guaraim and BRS Marcante. Our result agrees with [13], which says that 60% to 90% of the phytochemical compounds in cereals occur in a bound form.

## 3. Materials and Methods

### 3.1. Wheat Samples

Wheat (*T. aestivum* L.) grains of BRS Marcante and BRS Guaraim cultivars were obtained in the experimental fields of the Brazilian Agricultural Research Corporation (Embrapa Trigo) in Passo Fundo, RS state, Brazil (geographical coordinates: 28°15′46″ S; 52°24′24″ W; 687 m), in two crop seasons.

### 3.2. Samples Characterization

The GHI (method 55-31.01, AACCI [25], Perten Instruments, Springfield, IL, USA), grain falling number, (GFN, method 56-81.03, AACCI [25], Perten Instruments, Springfield, IL, USA), hectoliter weight (method 55-10.01, AACCI [25] with results expressed in kg·hL^−1^) and thousand kernel weight [60] were assessed to characterize the grains. The technological quality of wheat cultivars was evaluated according to AACCI [25] and included alveography (method 54-30.02, AACCI [25], Chopin Alveograph, Villeneuve-la-Garenne, France) of whole wheat flours, considering the parameters gluten strength (W), tenacity (P) and tenacity/extensibility ratio (P/L).

### 3.3. Germination

Wheat grain germination was performed as described by Baranzelli et al. [2] with some modification. Wheat grains were germinated in triplicate under controlled lighting conditions (12 h day/12 h night), relative humidity (80%), grain moisture (30%) and temperature (25 °C day/15 °C night) for0, 24, 48, and 72 h. Germination temperatures were selected according to the average of the maximum (day) and minimum (night) temperatures of the years 2014, 2015 and 2016 in Passo Fundo during the wheat crop season (September to November). This region is among the major Brazilian wheat producers.

### 3.4. Grain Milling

The whole wheat grains were first ground in a knife mill (Marconi, Piracicaba, SP, Brazil), followed by grinding in a hammer mill (Perten Instruments, Springfield, IL, USA) with a 0.8 mm sieve.

### 3.5. Germination Marker

The GFN was evaluated according to method 56–81.03 (AACCI [25], Perten Instruments, Springfield, IL, USA) with altitude correction (Passo Fundo, RS, Brazil = 687 m).

### 3.6. Extraction of Free and Matrix-Bound Phytochemical Compounds

Free phytochemical compounds (FPC) were extracted from 0.25 g of whole wheat flour diluted in 2.5 mL of acetone/water (80:20, *v*/*v*) solution acidified with 0.1% formic acid. Samples were vortex-mixed for 2 min, centrifuged at 1500× *g* for 3 min and the supernatant was collected. Extraction was repeated three times, the supernatants were pooled and 6 mL of extract was concentrated in a rotary evaporator at 38 °C.

Matrix-bound phytochemical compounds (BPC) were extracted through alkaline and acid hydrolysis according to Zhang et al. [61] with adaptations. For alkaline hydrolysis, 2.0 mL of distilled water and 1.5 mL of 3 mol·L^−1^ NaOH were added to the residue remaining after FPC extraction. Samples were shaken for 16 h at 20 °C and then had the pH adjusted to ~2.0. Phytochemical compounds released were extracted three times with 15 mL of diethyl ether/ethyl acetate solution (1:1, *v*/*v*). The supernatants were mixed, and 40 mL was dried in a rotary evaporator at 38 °C. The acid hydrolysis was carried out by adding 1.5 mL of 6 mol·L^−1^ HCl to the residue remaining after alkaline hydrolysis. Samples were incubated at 85 °C for 30 min and, thereafter, had the pH adjusted to ~2.0. The extraction followed the same steps as described for alkaline hydrolysis.

Extracts of FPC and BPC obtained through alkaline and acidic hydrolysis were resuspended in 1.2 mL of acidified water/methanol solution (1:0.2 *v*/*v*, 0.1% formic acid) and stored at −20 °C until further analysis.

### 3.7. Phytochemical Compounds Identification

The phytochemical compounds of wheat extracts were identified using two different sets of liquid chromatograph (LC) equipment. The first exploratory analysis was conducted using an LC system connected to a mass spectrometer (MS) that had a hybrid Orbitrap analyzer and an electrospray ionization source (ESI, Q-ExactiveTM, Thermo Fisher Scientific, Bremen, Germany), using the chromatographic conditions previously described by Astudillo-Pascual et al. [62].

Thereafter, compound identification was accomplished using an LC-MS equipped with a quadrupole-time-of flight (Q-TOF) analyzer and an electrospray ionization source (ESI) (Bruker Daltonics, micrOTOF-Q III model, Bremen, Germany). Chromatographic parameters were set according to Quatrin et al. [63] and Q-TOF-MS parameters were set according to Mallmann et al. [64].

The identification of phytochemical compounds was based on the retention time and order of elution in the reverse phase column, maximum absorption wavelength (UV-vis) and characteristics of the MS spectrum compared to authentic standards analyzed under the same conditions or with literature data. For the reliable identification of compounds, comparison was made with analytical standards and databases, based on neutral mass isotope distribution, retention time and MS/MS fragments using the customized database of polyphenols from PubChem (https://pubchem.ncbi.nlm.nih.gov/ (accessed on 20 July 2020)) and other metabolites from KEGG (http://www.genome.jp/kegg/ (accessed on 23 July 2020)), MoNA: MassBank of North America (https://mona.fiehnlab.ucdavis.edu/ (accessed on 3 August 2020)), FooDB (https://foodb.ca (accessed on 12 August 2020)) and ReSpect for Phytochemicals (http://spectra.psc.riken.jp/menta.cgi/respect/index (accessed on 13 August 2020)). For the non-targeted identification, the parameters generated by the software were applied in descending order of importance: precursor exact mass error and fragment mass error (<10 ppm); isotopic similarity (>80%) and highest fragmentation score (score > 30).

### 3.8. Quantification of Phytochemical Compounds

The quantification of free and matrix-bound phytochemical compounds of germinated wheat samples was performed according to Quatrin et al. [63].

Absorption spectra were recorded from 200 to 800 nm, and chromatograms for the quantification of phytochemical compounds were obtained at 280, 320 and 360 nm. Results were expressed as mg·100 g^−1^ of whole wheat flour on dry basis. The quantification conditions were validated in our laboratory (Nidal-UFSM) (Supplementary materials, Box S1 and Table S5) following the International Conference on Harmonization Guidelines.

### 3.9. Oxygen Radical Absorbance Capacity (ORAC)

The oxygen radical absorbance capacity (ORAC) of FPC was determined according to the method described by Ou, Hampsch-woodill and Prior [65]. Values were expressed as µmol of Trolox equivalents.g^−1^ sample on dry basis.

### 3.10. Statistical Analysis

The results of germination markers and ORAC were analyzed using 2-way analysis of variance (ANOVA, 2 cultivars × 4 germination times) with six replicates (n = 6, where three replicates each were provided from two different crop seasons), followed by Tukey’s test for mean comparison at a level of 5% significance. Statistica software (version 9.0, StatSoft Inc., Tulsa, OK, USA) was used to perform this statistical analysis.

For the results of the phytochemical quantification, the experimental design was randomized into blocks in a 2 × 4 factorial scheme (2 wheat cultivars × 4 germination times), totaling eight (8) treatments with six replicates each (n = 6). Data were submitted to ANOVA using the GLM procedure, their means were adjusted using the ordinary least squares method with the LSMEANS command and compared using the Student–Newman–Keuls (SNK) test.

Then, multivariate analysis of variance (MANOVA) was performed, in which the matrices of sums of squares and products were obtained. To test the hypothesis that the treatment means vectors were null, the Wilks (λ), Pillai (V), Hotelling–Lawley (U) and Roy (F_0_) tests were performed. Additionally, cluster analysis was performed for treatments using the DISTANCE, CLUSTER and TREE procedures, using the average Euclidean distance as a dissimilarity measure and Ward as the clustering method. A cluster analysis of the dependent variables was also performed using the VARCLUS and TREE procedures, using the correlation matrix as input. Principal component analysis was performed using the PRINQUAL, PRINCOMP and FACTOR procedures (Ravindra Khattree and Dayanand N. Naik 2000). Statistical analyses were performed on the SAS^®^ System for Windows^™^ version 9.4 (SAS Institute Inc., Cary, NC, USA), at a 5% significance level.

Chromatogram figures were obtained from the LabSolutions software, dendogram figures were obtained from the SAS^®^ software, PCA figures were produced using Microsoft Excel and the antioxidant capacity figure was produced using GraphPad Prism version 6 for Windows (GraphPad Software, San Diego, CA, USA).

## 4. Conclusions

Germination proved to be a good tool to increase and diversify the content of bioactive compounds in wheat, as indicated by the increase in the content of benzoxazinoids and antioxidant capacity. Changes in the profile of phytochemical compounds during germination were different between the soft- and hard-texture cultivars, and the hard-texture cultivar had the greatest increase in antioxidant capacity after germination. Before germination, the hard-texture cultivar, BRS Marcante, had a higher content of total benzoxazinoids and flavonoids than the soft-texture cultivar, BRS Guaraim, including DIBOA-hex-hex, free and bound sinapic acid, and apigenins hex-pent II, and hex-hex-hex I and II. On the other hand, the soft-texture cultivar, BRS Guaraim, had a greater content of DIMBOA-hex-hex. Free phytochemical compounds, mainly benzoxazinoids, increased during germination in both cultivars. Before germination, few differences were observed between the soft and hard cultivar concerning the profile of matrix-bound phytochemicals, which comprise the major group of phenolic compounds in wheat. During germination, the levels of this group of compounds have been shown to decrease only in the hard-texture cultivar, due to decreased levels of phenolic acids (*trans*-ferulic acid) and flavonoids (apigenin) that were bound to the cell wall through ester-type bonds. These findings confirm the hypothesis that hard and soft wheat cultivars have distinct behavior during germination concerning the changes in phytochemical compounds, namely the matrix-bound compounds. In addition, germination has been shown to remarkably increase the content of benzoxazinoids, and the antioxidant capacity of the free phytochemical fraction of wheat, which could bring a health-beneficial appeal for pre-harvested sprouted grains that have lost their baking properties.

## Figures and Tables

**Table 1:** Germination follow-up through grain falling number values (GFN, expressed in seconds) of wheat cultivars from soft (BRS Guaraim) and hard (BRS Marcante) texture.

Germination Time	BRS Guaraim	BRS Marcante	Time Mean
0 h	352 ± 12 ^aB^	389 ± 6 ^aA^	370 ± 8
24 h	216 ± 7 ^bA^	162 ± 7 ^bB^	189 ± 9
48 h	96 ± 5 ^cA^	69 ± 2 ^cB^	82 ± 4
72 h	62 ± 0 ^dA^	62 ± 0 ^cA^	62 ± 0
Cultivar mean	181 ± 21	171 ± 24	

Means ± standard error of mean were reported (n = 6). Values that have no common superscript letter are significantly different (*p* < 0.05) within the same row (uppercase letters) or within the same column (lowercase letter).

**Table 2:** Changes in free phytochemical compounds (mg·100 g^−1^ whole wheat flour d.b.) of wheat grains according to the germination time (G) and cultivar (C).

Cultivar (C)	Germination Time (G)				Mean	*p*-Value		
	0 h	24 h	48 h	72 h		C	G	C × G
	Vanillic acid					0.1977	0.0233	0.0035
BRS Guaraim	0.18 ^Aa^	0.00 ^b^	0.00 ^b^	0.00 ^b^	0.04			
BRS Marcante	0.00 ^B^	0.00	0.00	0.06	0.02			
Mean	0.09	0.00	0.00	0.03			SEM =	0.01330
	DIBOA-hex-hex *					0.0006	0.0001	0.7841
BRS Guaraim	1.39	1.50	4.09	10.28	4.31 ^B^			
BRS Marcante	3.97	3.97	8.66	14.63	7.81 ^A^			
Mean	2.68 ^c^	2.73 ^c^	6.37 ^b^	12.45 ^a^			SEM =	0.79561
	DIMBOA-hex-hex *					0.7277	0.0001	0.8312
BRS Guaraim	0.69	0.54	3.64	7.61	3.12			
BRS Marcante	0.79	0.95	2.75	7.23	2.93			
Mean	0.74 ^c^	0.74 ^c^	3.19 ^b^	7.42 ^a^			SEM =	0.47198
	Apigenin-hex-pent I ^#^					0.048	0.0159	0.0414
BRS Guaraim	4.05 ^a^	2.29 ^Bb^	4.06 ^a^	4.62 ^a^	3.75			
BRS Marcante	4.16	4.25 ^A^	4.38	4.47	4.31			
Mean	4.10	3.27	4.22	4.54			SEM =	0.16399
	Sinapic acid					0.0001	0.8075	0.9454
BRS Guaraim	0.50	0.49	0.52	0.47	0.49 ^B^			
BRS Marcante	0.92	0.93	0.89	0.85	0.89 ^A^			
Mean	0.71	0.71	0.70	0.66			SEM =	0.03594
	Apigenin-hex-pent II ^#^					0.0001	0.937	0.3065
BRS Guaraim	6.27	6.49	6.37	6.79	6.48 ^B^			
BRS Marcante	7.77	7.75	7.63	7.43	7.65 ^A^			
Mean	7.02	7.12	7.00	7.11			SEM =	0.11631
	Apigenin-hex-hex-hex I ^#^					0.0001	0.0347	0.5372
BRS Guaraim	4.11	4.22	4.75	4.49	4.39 ^B^			
BRS Marcante	5.14	5.19	5.38	5.23	5.23 ^A^			
Mean	4.62 ^b^	4.70 ^ab^	5.06 ^a^	4.86 ^ab^			SEM =	0.08466
	Apigenin-hex-hex-hex II ^#^					0.0001	0.101	0.2309
BRS Guaraim	5.20	5.53	5.44	6.48	5.66 ^B^			
BRS Marcante	8.38	8.60	8.92	8.70	8.65 ^A^			
Mean	6.79	7.06	7.18	7.59			SEM =	0.25354
	Apigenin ^#^					0.8968	0.0001	0.9937
BRS Guaraim	3.89	3.99	1.94	3.83	3.41			
BRS Marcante	3.91	3.83	1.92	3.86	3.38			
Mean	3.90 ^a^	3.91 ^a^	1.93 ^b^	3.84 ^a^			SEM =	0.18772
	Sum of free phenolic acids					0.0001	0.3897	0.4308
BRS Guaraim	0.68	0.49	0.52	0.47	0.54 ^B^			
BRS Marcante	0.92	0.93	0.89	0.91	0.91 ^A^			
Mean	0.8	0.71	0.7	0.69			SEM =	0.03671
	Sum of free benzoxazinoids					0.022	0.0001	0.9849
BRS Guaraim	2.08	2.03	7.72	17.88	7.43 ^B^			
BRS Marcante	4.76	4.92	11.41	21.86	10.74 ^A^			
Mean	3.42 ^c^	3.48 ^c^	9.56 ^b^	19.87 ^a^			SEM =	1.22276
	Sum of free flavonoids					0.0001	0.0368	0.2241
BRS Guaraim	23.5	22.5	22.6	26.2	23.7 ^B^			
BRS Marcante	29.4	29.6	28.2	29.7	29.2 ^A^			
Mean	26.4 ^ab^	26.1 ^ab^	25.4 ^b^	27.9 ^a^			SEM =	0.53165
	Sum of free phytochemical compounds					0.0001	0.0001	0.9583
BRS Guaraim	26.3	25.0	30.8	44.5	31.7 ^B^			
BRS Marcante	35.0	35.5	40.5	52.4	40.9 ^A^			
Mean	30.7 ^c^	30.2 ^c^	35.7 ^b^	48.5 ^a^			SEM =	1.55007

Means (n = 6) followed by different capital letters within the same column and different small letters within the same row differ (*p* < 0.05), respectively, between cultivars and germination times using the Student-–Newman-–Keuls test. * Benzoxazinoids were quantified as equivalent to 2H-1,4-benzoxazin-3(H)-one; # Flavonoids were quantified as equivalent to quercetin. DIBOA: 2,4-dihydroxy-1,4-benzoxazin-3-one; DIMBOA: Dihydroxy-7-methoxy-1,4-benzoxazin-3-one; hex: hexoside; pent: pentoside; LoQ: limit of quantification; SEM: standard error of mean. Vanillic acid LoQ: 0.025 ppm; 2H-1,4-benzoxazin-3(H)-one LoQ: 0.106 ppm; Quercetin LoQ: 0.444 ppm; Sinapic acid LoQ: 0.258 ppm.

**Table 3:** Changes in bound phytochemical compounds (mg·100 g^−1^ whole wheat flour d.b.) of wheat grains according to the germination time (G) and cultivar (C).

Cultivar (C)	Germination Time (G)				Mean	*p-*Value		
	0 h	24 h	48 h	72 h		C	G	C × G
Bound phytochemical compounds—Alkaline hydrolysis								
	4-Hydroxybenzoic acid					0.489	0.0008	0.003
BRS Guaraim	0.27	0.61	1.03 ^B^	1.64 ^A^	0.89			
BRS Marcante	0.00 ^b^	0.00 ^b^	2.71 ^Aa^	0.00 ^Bb^	0.68			
Mean	0.14	0.30	1.87	0.82			SEM =	0.19072
	Vanillic acid					0.029	0.0044	0.004
BRS Guaraim	0.00 ^b^	0.00 ^b^	0.09 ^Aa^	0.00 ^b^	0.02			
BRS Marcante	0.00	0.00	0.00 ^B^	0.00	0.00			
Mean	0.00	0.00	0.05	0.00			SEM =	0.00673
	*p*-Coumaric acid					0.317	0.1711	0.374
BRS Guaraim	2.85	2.97	3.03	2.76	2.90			
BRS Marcante	3.24	2.93	3.04	2.78	3.00			
Mean	3.04	2.95	3.04	2.77			SEM =	0.04886
	*trans*-Ferulic acid					0.61	0.2318	0.027
BRS Guaraim	44.2	45.7	50.9 ^A^	43.9	46.2			
BRS Marcante	56.5 ^a^	50.8 ^a^	27.9 ^Bb^	40.9 ^ab^	44.0			
Mean	50.3	48.3	39.4	42.4			SEM =	2.24618
	Sinapic acid					0.046	0.0143	0.319
BRS Guaraim	3.80	4.70	5.82	4.24	4.64 ^B^			
BRS Marcante	5.68	5.44	6.15	4.30	5.39 ^A^			
Mean	4.74 ^ab^	5.07 ^ab^	5.99 ^a^	4.27 ^b^			SEM =	0.20523
	Apigenin ^#^					0.016	0.0015	0.004
BRS Guaraim	11.0	11.0	11.1	10.8 ^A^	11.0			
BRS Marcante	11.0 ^a^	10.8 ^a^	11.0 ^a^	5.4 ^Bb^	9.5			
Mean	11.0	10.9	11.0	8.1			SEM =	0.38845
	Sum of bound phenolic acids—Alkaline hydrolysis					0.734	0.461	0.0463
BRS Guaraim	51.1	54.0	60.9 ^A^	52.5	54.6			
BRS Marcante	65.4 ^a^	59.2 ^ab^	39.8 ^Bb^	48.0 ^ab^	53.1			
Mean	58.2	56.6	50.4	50.2			SEM =	2.33542
	Sum of bound flavonoids—Alkaline hydrolysis					0.016	0.002	0.0035
BRS Guaraim	11.0	11.0	11.1	10.8 ^A^	11.0			
BRS Marcante	11.0 ^a^	10.8 ^a^	11.0 ^a^	5.4 ^Bb^	9.5			
Mean	11.0	10.9	11.0	8.1			SEM =	0.38845
	Sum of bound phytochemical compounds—Alkaline hydrolysis					0.522	0.309	0.0449
BRS Guaraim	62.1	65.0	72.0 ^A^	63.3	65.6			
BRS Marcante	76.4 ^a^	70.0 ^ab^	50.8 ^Bb^	53.3 ^b^	62.6			
Mean	69.2	67.5	61.4	58.3			SEM =	2.43190
Bound phytochemical compounds—Acid hydrolysis								
	*p*-Coumaric acid					0.9534	0.5427	0.0309
BRS Guaraim	8.56	7.47 ^B^	9.39	8.72	8.53			
BRS Marcante	7.73	9.36 ^A^	8.11	9.04	8.56			
Mean	8.14	8.41	8.75	8.88			SEM =	0.20815

Means (n = 6) followed by different capital letters within the same column and different small letters within the same row differ (*p* < 0.05), respectively, between cultivars and germination times using the Student–Newman–Keuls test. # Flavonoids were quantified as equivalent to quercetin. LoQ: limit of quantification; SEM: standard error of mean. 4-Hydroxybenzoic acid LoQ: 0.062; Vanillic acid LoQ: 0.025 ppm; *p*-Coumaric acid LoQ: 0.029 ppm; *trans*-Ferulic acid LoQ: 0.033 ppm; Sinapic acid LoQ: 0.258 ppm; Quercetin LoQ: 0.444 ppm.

## Data Availability

The data presented in this study are available on request from the corresponding author.

## Supplementary Materials

The following supporting information can be downloaded at: https://www.mdpi.com/article/10.3390/molecules28020721/s1. Table S1. Technological characteristics of two Brazilian wheat cultivars bearing soft (BRS Guaraim) and hard (BRS Marcante) texture grains. Table S2. Grain images during germination. Figure S1. Representative LC-PDA chromatograms of phytochemicals extracted from wheat grains. Free compounds (a) and bound compounds obtained by alkaline (b) and acid (c) hydrolysis. Table S3. Tentative identification of wheat phytochemicals by LC-Q-TOF-MS and LC-Orbitrap-MS. Table S4. Results of multivariate analysis of variance (MANOVA) for phytochemical compounds from wheat cultivars (C) under different germination times (T), using likelihood ratio test (Wilks) and Pillai, Hotelling-Lawley and Roy’s tests. Box S1. Validation data for the analysis of benzoxazinone and phenolic compounds. Table S5. Accuracy test for the analysis of phenolic and benzoxazinoid compounds in wheat grains.
