# Supplementary material for: Grain Germination Changes the Profile of Phenolic Compounds and Benzoxazinoids in Wheat: A Study on Hard and Soft Cultivars
Source: Molecules. 2023 Jan 11;28(2):721. doi: 10.3390/molecules28020721 (PMC9864386; doi:10.3390/molecules28020721)
Supplement: Supplementary file 1 [file molecules-28-00721-s001.zip › molecules-2127641-supplementary.pdf]

Supplementary material

Grain germination changes the profile of phenolic compounds and benzoxazinoids in wheat: a study on hard and soft cultivars

Julia Baranzelli[1], Sabrina Somacal[1], Camila Sant'Anna Monteiro[1], Renius de Oliveira Mello[1], Eliseu Rodrigues[1], Osmar Damian Prestes[3], Rosalía López-Ruiz[4], Antonia Garrido Frenich[4], Roberto Romero-González[4], Martha Zavariz de Miranda[5], Tatiana Emanuelli[1,*]

[1] Department of Food Technology and Science, Center of Rural Sciences, Federal University of Santa Maria, 97105-900, Santa Maria, RS, Brazil; julia.baranzelli@acad.ufsm.br; sabrina.somacal@ufsm.br; camila.monteiro@acad.ufsm.br; renius.mello@ufsm.br; tatiana.emanuelli@ufsm.br
[2] Department of Food Science, Federal University of Rio Grande do Sul, 91501-970, Porto Alegre, RS, Brazil; eliseu.rodrigues@ufrgs.br
[3] Department of Chemistry, Center of Natural and Exact Sciences, Federal University of Santa Maria, 97105-900, Santa Maria, RS, Brazil; osmar.prestes@ufsm.br
[4] Research Group 'Analytical Chemistry of Contaminants', Department of Chemistry and Physics, Research Centre for Mediterranean Intensive Agrosystems and Agri-Food Biotechnology (CIAIMBITAL), University of Almeria, 04120, Almeria, Spain; rlr468@ual.es; agarrido@ual.es; rromero@ual.es
[5] Grain Quality Laboratory, Brazilian Agricultural Research Corporation - Embrapa Trigo, 99050-970, Passo Fundo, RS, Brazil; martha.miranda@embrapa.br

* Correspondence: tatiana.emanuelli@ufsm.br

Table S1. Technological characteristics of two Brazilian wheat cultivars bearing soft (BRS Guaraim) and hard (BRS Marcante) texture grains.

	**BRS Guaraim**	**BRS Marcante**
Grain		
Hardness index (GHI)	35.3 ± 0.6^{b}	74.5 ± 0.6^{a}
Falling number (GFN, s)	362 ± 2^{b}	589 ± 13^{a}
Hectoliter weight (HW, kg. hL^{-1})	72.8 ± 0.0^{b}	79.0 ± 0.0^{a}
Thousand kernel weight (TKW, g)	31.3 ± 0.3^{b}	35.3 ± 0.0^{a}
Whole wheat flour		
**Alveography**		
Gluten strength (W x 10^{-4} J)	96 ± 4^{b}	254 ± 6^{a}
Tenacity (P, mm Hg)	112 ± 1^{b}	226 ± 5^{a}
Tenacity/extensibility ratio (P/L)	5.7 ± 0.2^{b}	8.5 ± 0.3^{a}

*Different letters indicate significant differences within the same line ($p<0.05$). Analysis were performed in duplicate and the means ± standard deviation were reported.

Table S2. Grain images during germination.

Germ. time	BRS Guaraim		BRS Marcante	
	Crop season 1 (2015)	Crop season 2 (2016)	Crop season 1 (2013)	Crop season 2 (2016)
0h	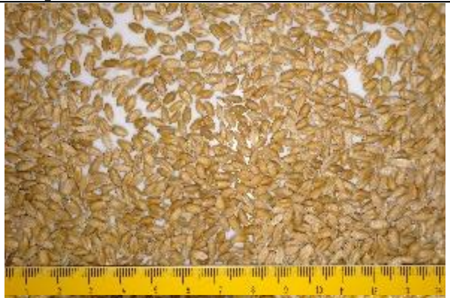	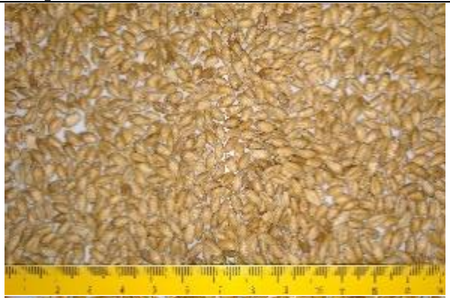	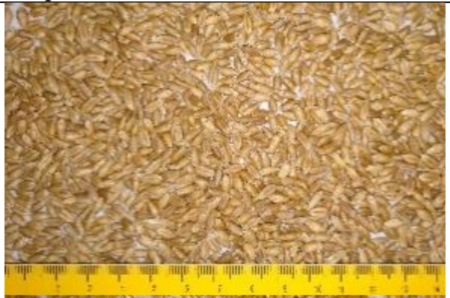	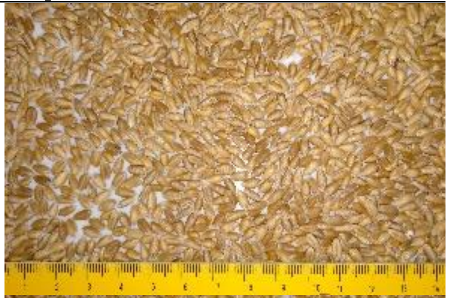
24h	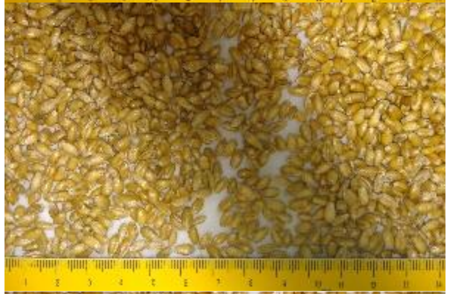	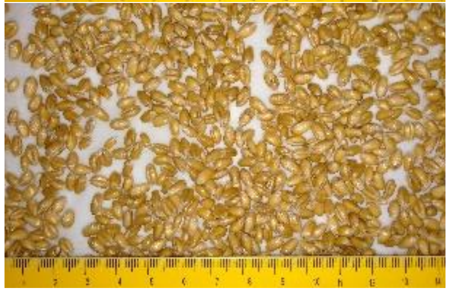	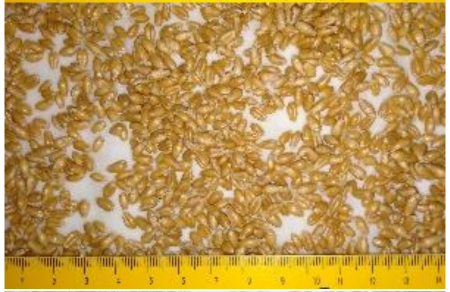	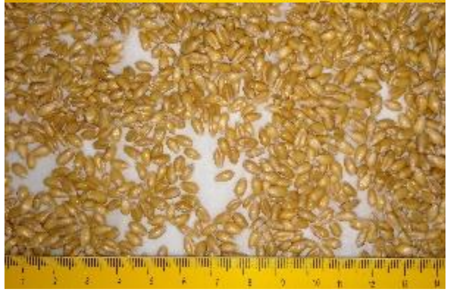
48h	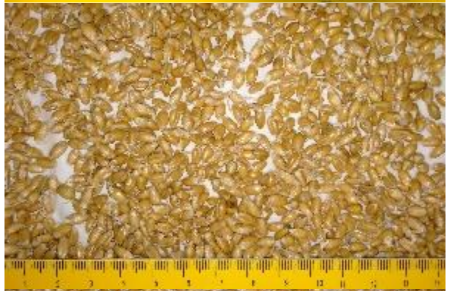	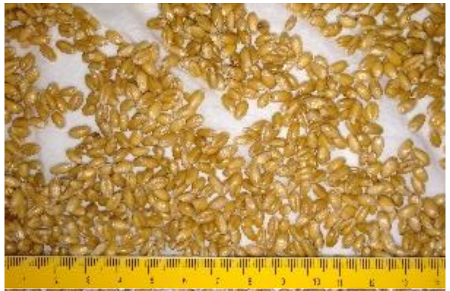	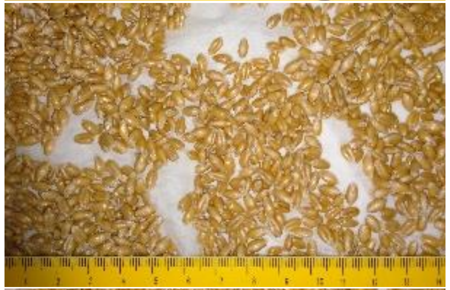	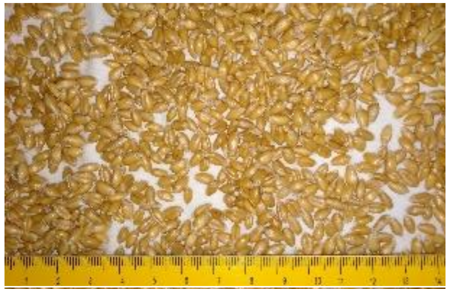
72h	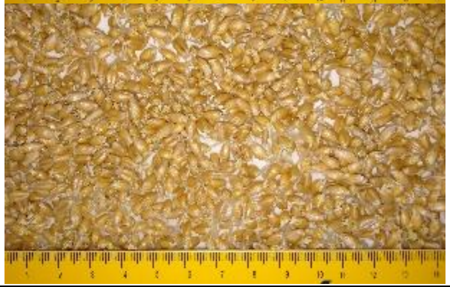	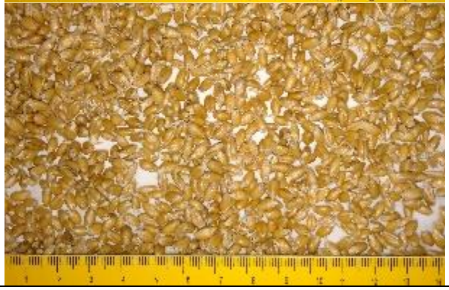	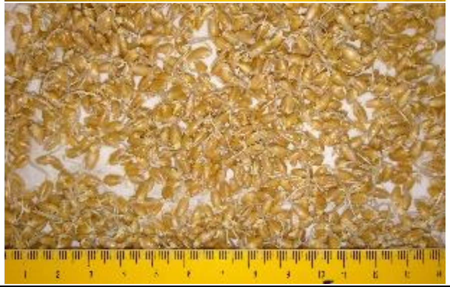	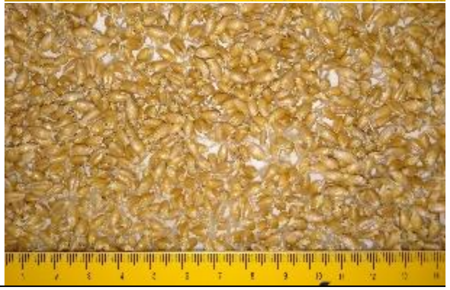

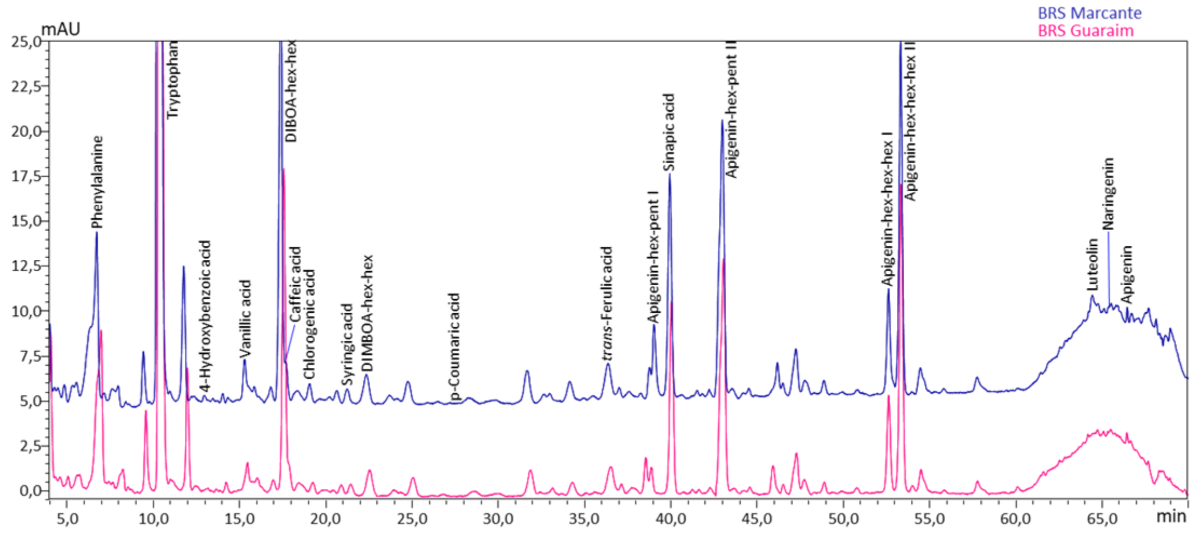

(**a**)

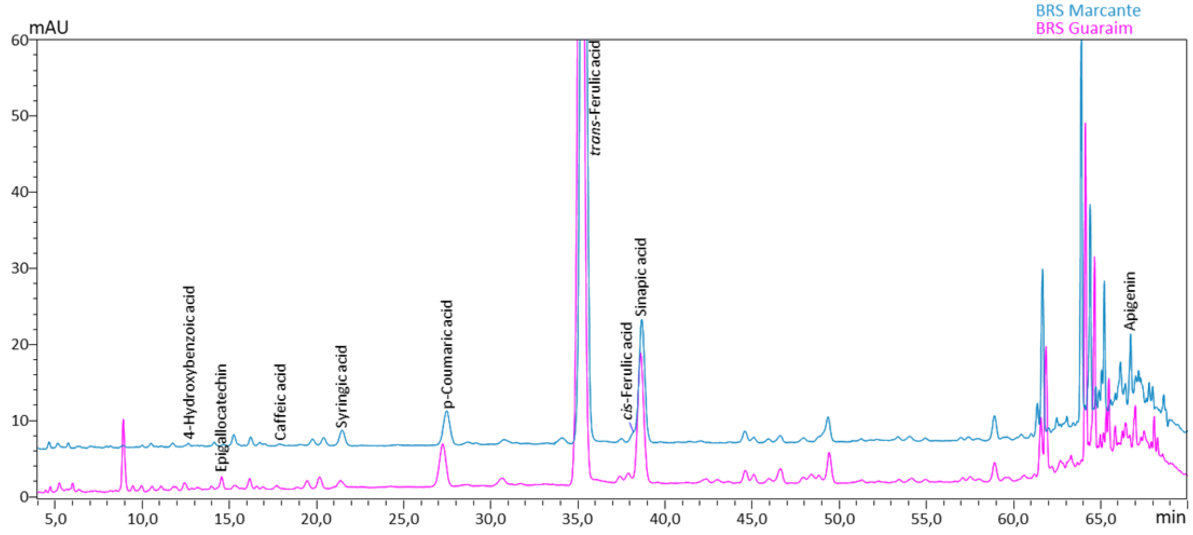

(**b**)

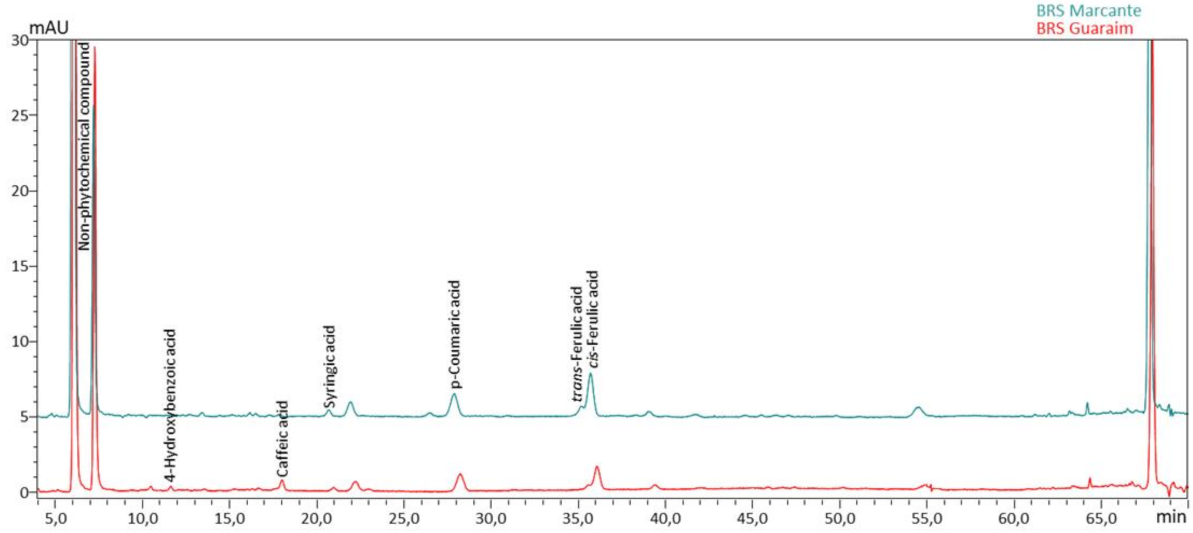

(**c**)

Figure S1. Representative LC-PDA chromatograms of phytochemicals extracted from wheat grains. Free compounds (**a**) and bound compounds obtained by alkaline (**b**) and acid (**c**) hydrolysis.

Table S3. Tentative identification of wheat phytochemicals by LC-Q-TOF-MS and LC-Orbitrap-MS.

nº peak	RT (min)	λ (nm)	λ_{max} (nm)	Tentative identification	Compound class	Compound sub-class	Molecular formula	Monoisotopic mass	Experimental mass	MS/MS fragments	Ionization/ Polarity	Method identification	Reference
**Free phytochemical compounds**													
1	6.3	280	282	Phenylalanine	Amino acid		$C_9H_{11}NO_2$	165.0790	165.0822	103.0590 (100); 120.0851 (65); 104.0620 (12)	$[M+H]^+$	LC-Q-TOF-MS	[35]
2	10.3	280	278	Tryptophan	Amino acid		$C_{11}H_{12}N_2O_2$	204.0899	204.0940	118.0717 (100); 143.0776 (42); 146.0636 (28); 144.0827 (25); 115.0588 (23)	$[M+H]^+$	LC-Q-TOF-MS	[35]
3	12.2	280	275, 260	4-Hydroxybenzoic acid[a]	Phenolic acid	HBD	$C_7H_6O_3$	138.0317	n.d.	n.d.	Negative	LC-Orbitrap-MS	
4	16.6	280	260, 292	Vanillic acid[a]	Phenolic acid	HBD	$C_8H_8O_4$	168.0423	167.0350	n.d.	Negative	LC-Orbitrap-MS	
5	17.2	280	254, 278	DIBOA-hex-hex	BX	Hydroxamic acid	$C_{20}H_{27}NO_{14}$	505.1432	505.1208	134.0273 (100); 162.0220 (40); 174.0586 (4)	$[M-H]^-$	LC-Q-TOF-MS	[36,37]
6	17.5	320	322	Caffeic acid[a]	Phenolic acid	HCD	$C_9H_8O_4$	180.0423	n.d.	n.d.	Negative	LC-Orbitrap-MS	
7	17.6	320	325	Chlorogenic acid[a]	Phenolic acid	HCD	$C_{16}H_{18}O_9$	354.0951	355.1024	n.d.	Positive	LC-Orbitrap-MS	
8	24.3	280	252, 275	DIMBOA-hex-hex	BX	Hydroxamic acid	$C_{21}H_{29}NO_{15}$	535.1537	535.1543	164.0376 (100); 149.0126 (71); 192.0317 (12)	$[M-H]^-$	LC-Q-TOF-MS	[37]
9	27.8	320	308	p-Coumaric acid[a]	Phenolic acid	HCD	$C_9H_8O_3$	164.0473	n.d.	n.d.	Negative	LC-Orbitrap-MS	
10	35.7	320	321	*trans*-Ferulic acid[a]	Phenolic acid	HCD	$C_{10}H_{10}O_4$	194.0579	n.d.	n.d.	Negative	LC-Orbitrap-MS	
11	38.5	320	275, 334	Apigenin-hex-pent I	Phenolic acid	Flavone	$C_{26}H_{28}O_{14}$	564.1480	564.1439	563.1309 (100); 443.0934 (45); 473.1026 (34);	$[M-H]^-$	LC-Q-TOF-MS	[38]

nº peak	RT (min)	λ (nm)	λ_{max} (nm)	Tentative identification	Compound class	Compound sub-class	Molecular formula	Monoisotopic mass	Experimental mass	MS/MS fragments	Ionization/ Polarity	Method identification	Reference
										353.0628 (30); 383.0727 (22)			
12	39.1	320	333, 271	Sinapic acid[a]	Phenolic acid	HCD	$C_{11}H_{12}O_5$	224.0685	223.0612	n.d.	Negative	LC-Orbitrap-MS	
13	42.8	320	270, 336	Apigenin-hex-pent II	Flavonoid	Flavone	$C_{26}H_{28}O_{14}$	564.1479	564.1432	443.0946 (39); 353.0658 (37); 383.0761 (34); 473.1056 (32)	$[M-H]^-$	LC-Q-TOF-MS	[38]
14	52.4	320	332, 272	Apigenin-hex-hex-hex I	Flavonoid	Flavone	$C_{33}H_{38}O_{21}$	770.1906	770.1912	425.0824 (100); 545.1224 (79); 426.0868 (26); 546.1238 (23)	$[M-H]^-$	LC-Q-TOF-MS	MoNA
15	53.1	320	330, 272	Apigenin-hex-hex-hex II	Flavonoid	Flavone	$C_{33}H_{38}O_{21}$	770.1906	770.1916	425.0824 (100); 545.1222 (97); 546.1251 (27); 426.0860 (25)	$[M-H]^-$	LC-Q-TOF-MS	[39]
16	64.1	360	348	Luteolin[a]	Flavonoid	Flavone	$C_{15}H_{10}O_6$	286.0477	n.d.	n.d.	Negative	LC-Orbitrap-MS	
17	65.5	280	288	Naringenin[a]	Flavonoid	Flavanona	$C_{15}H_{12}O_5$	272.0685	271.0612	n.d.	Negative	LC-Orbitrap-MS	
18	66.5	320	324, 281	Apigenin[a]	Flavonoid	Flavone	$C_{15}H_{10}O_5$	270.0528	271.0601	n.d.	Positive	LC-Orbitrap-MS	
**Bound phytochemical compounds – Alkaline hydrolysis**													
1	12.2	280	275, 260	4-Hydroxybenzoic acid[a]	Phenolic acid	HBD	$C_7H_6O_3$	138.0317	n.d.	n.d.	Negative	LC-Orbitrap-MS	
2	13.9	280	270	Epigallocatechin	Flavonoid	Flavanol	$C_{15}H_{14}O_7$	306.0739	305.0667	n.d.	Negative	LC-Orbitrap-MS	
3	17.5	320	322	Caffeic acid[a]	Phenolic acid	HCD	$C_9H_8O_4$	180.0423	n.d.	n.d.	Negative	LC-Orbitrap-MS	

nº peak	RT (min)	λ (nm)	λ_{max} (nm)	Tentative identification	Compound class	Compound sub-class	Molecular formula	Monoisotopic mass	Experimental mass	MS/MS fragments	Ionization/ Polarity	Method identification	Reference
4	21.0	280	274	Syringic acid[a]	Phenolic acid	HBD	$C_9H_{10}O_5$	198.0528	197.0456	n.d.	Negative	LC-Orbitrap-MS	
5	27.8	320	308	*p*-Coumaric acid[a]	Phenolic acid	HCD	$C_9H_8O_3$	164.0473	164.0478	**119.0503** (20)	$[M-H]^-$	LC-Q-TOF-MS and LC-Orbitrap-MS	
6	35.7	320	321	*trans*-Ferulic acid[a]	Phenolic acid	HCD	$C_{10}H_{10}O_4$	194.0579	194.0587	133.0296 (100); 134.0372 (79)	$[M-H]^-$	LC-Q-TOF-MS and LC-Orbitrap-MS	
7	36.7	320	316	*cis*-Ferulic acid	Phenolic acid	HCD	$C_{10}H_{10}O_4$	194.0579	194.0586	134.0374 (100); 133.0287 (82)	$[M-H]^-$	LC-Q-TOF-MS	
8	39.1	320	333, 271	Sinapic acid[a]	Phenolic acid	HCD	$C_{11}H_{12}O_5$	224.0685	223.0612	n.d.	Negative	LC-Orbitrap-MS	
**Bound phytochemical compounds – Acid hydrolysis**													
1	12.2	280	275, 260	4-Hydroxybenzoic acid[a]	Phenolic acid	HBD	$C_7H_6O_3$	138.0317	137.0317	n.d.	Negative	LC-Orbitrap-MS	
2	17.5	320	322	Caffeic acid[a]	Phenolic acid	HCD	$C_9H_8O_4$	180.0423	179.0423	n.d.	Negative	LC-Orbitrap-MS	
3	21.0	280	274	Syringic acid[a]	Phenolic acid	HBD	$C_9H_{10}O_5$	198.0528	197.0528	n.d.	Negative	LC-Orbitrap-MS	
4	27.8	320	308	*p*-Coumaric acid[a]	Phenolic acid	HCD	$C_9H_8O_3$	164.0473	163.0473	n.d.	Negative	LC-Orbitrap-MS	
5	35.7	320	321	*trans*-Ferulic acid[a]	Phenolic acid	HCD	$C_{10}H_{10}O_4$	194.0579	194.0582	133.0292 (100); 134.0368 (84)	$[M-H]^-$	LC-Q-TOF-MS and LC-Orbitrap-MS	
6	36.7	320	316	*cis*-Ferulic acid	Phenolic acid	HCD	$C_{10}H_{10}O_4$	194.0579	194.0579	n.d.	$[M-H]^-$	LC-Q-TOF-MS and LC-Orbitrap-MS	

[a] Positively identified by comparison with authentic standard. DIBOA: 2,4-dihydroxy-1,4-benzoxazin-3-one; DIMBOA: Dihydroxy-7-methoxy-1,4-benzoxazin-3-one; hex: hexoxide; pent: pentoxide.

Table S4. Results of multivariate analysis of variance (MANOVA) for phytochemical compounds from wheat cultivars (C) under different germination times (T), using likelihood ratio test (Wilks) and Pillai, Hotelling-Lawley and Roy's tests.

Tests	Sources of variation (*p*-value)		
	C	T	C×T
Free phytochemical compounds			
Wilks (λ)	0.0001	0.0001	0.0009
Pillai (V)	0.0001	0.0001	0.0005
Hotelling-Lawley (U)	0.0001	0.0001	0.0031
Roy (F_0)	0.0001	0.0001	0.0068
Bound phytochemical compounds – Alkaline hydrolysis			
Wilks (λ)	0.0105	0.0003	0.0001
Pillai (V)	0.0105	0.0007	0.0003
Hotelling-Lawley (U)	0.0105	0.0005	0.0001
Roy (F_0)	0.0105	0.0001	0.0001

Box S1. Validation data for the analysis of benzoxazinone and phenolic compounds.

Compound			Gallic acid[#]	Caffeic acid[#]	Benzoxazinone
λ (nm)			280	320	280
RT (min) (n= 3)			3.8	17.2	31.6
Linear range (mg.L^{-1})			0.04-60	0.02-60	0.11-60
Regression equation			y=79,089x+81,326	y=159,186x+120,861	y=46,478x-35,688
p**			0.81	0.96	0.98
R^2			0.998	0.999	0.999
Instrumental***		LoQ	0.012	0.006	0.106
		LoD	0.037	0.017	0.035
Repeatability intra-day precision, CV (%)	Low level (n= 3)	RT	0.50	0.04	0.20
		Peak area	4.84	6.46	1.90
	Medium level (n= 3)	RT	0.41	0.12	0.03
		Peak area	0.36	0.38	0.49
	High level (n= 3)	RT	0.21	0.05	0.05
		Peak area	1.80	0.93	0.33
Intermediate inter-day precision, CV (%) n= 10		RT	0.46	0.24	0.79
		Peak area	6.19	6.21	6.87

**p value for the lack of fitness; ***LoD and LoQ for the standard solution (mg.$^{L-1}$). RT: retention time; CV: coefficient of variation; Bx: 2H-1,4-Benzoxazin-3(H)-one; Low level: LoQ of the compound; Medium level: 30 ppm; High level: 60 ppm. [#]Data previously published by our group (Table S2 from Quatrin et al. [63]).

Table S5. Accuracy test for the analysis of phenolic and benzoxazinoid compounds in wheat grains.

Compounds	Accuracy (n=3)		
	Average recovery low level (%)*	Average recovery medium level (%)**	Average recovery high level (%)***
Gallic acid	102	105	84
Caffeic acid	99	88	109
2H-1,4-Benzoxazin-3(H)-one	86	85	74

*The low level was fortified with 80% of the amount of compounds present in the sample; **The medium level was fortified with 100% amount; ***The high level was fortified with 120% amount.
